# Dioxido{4,4′,6,6′-tetrabromo-2,2′-[2,2-dimethylpropane-1,3-diylbis(nitrilomethanylylidene)]diphenolato}molyb­denum(VI)

**DOI:** 10.1107/S1600536812039785

**Published:** 2012-09-26

**Authors:** Hadi Kargar, Muhammad Nawaz Tahir

**Affiliations:** a Department of Chemistry, Payame Noor University, PO Box 19395-3697, Tehran, I. R. of IRAN; b Department of Physics, University of Sargodha, Punjab, Pakistan

## Abstract

The asymmetric unit of the title compound, [Mo(C_19_H_16_Br_4_N_2_O_2_)O_2_], comprises two mol­ecules. The coordination environments around the Mo^VI^ atoms are distorted octa­hedral, defined by two oxide ligands and an N_2_O_2_ donor set of the tetra­dentate Schiff base in each mol­ecule. The dihedral angles between the benzene rings in the mol­ecules are 76.2 (3) and 77.7 (3)°. An inter­esting feature of the crystal structure is the presence of Br⋯Br contacts [3.4407 (11), 3.5430 (11) and 3.6492 (10) Å], which are shorter than the sum of the van der Waals radius of Br atoms (3.70 Å). The crystal structure is further stabilized by inter­molcular C—H⋯Br and C—H⋯π inter­actions. The crystal under investigation was twinned by nonmerohedry in a 0.053 (1):0.947 (1) ratio.

## Related literature

For the importance of molybdenum in molybdoenzymes, in coordination chemistry and in catalysis, see: Majumdar & Sarkar (2011 ▶); Enemark *et al.* (2004 ▶); Holm *et al.* (1996 ▶); Mancka & Plass (2007 ▶). For background to Schiff base ligands and their complexes with MoO_2_-containing units, see: Kia & Fun (2009 ▶); Kargar & Kia (2011 ▶). For related structures, see: Abbasi *et al.* (2008 ▶); Monadi *et al.* (2009 ▶). For van der Waals radii, see: Bondi (1964 ▶).
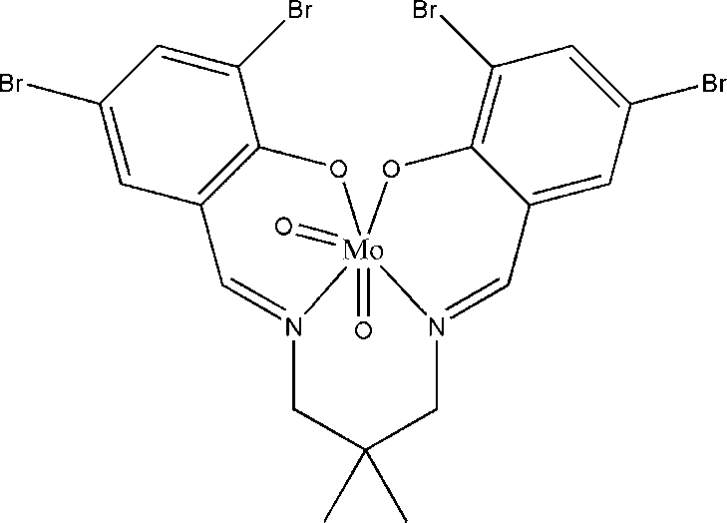


## Experimental

### 

#### Crystal data

[Mo(C_19_H_16_Br_4_N_2_O_2_)O_2_]
*M* *_r_* = 751.92
Monoclinic, 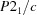

*a* = 13.1915 (6) Å
*b* = 15.7890 (8) Å
*c* = 22.2514 (13) Å
β = 101.702 (3)°
*V* = 4538.2 (4) Å^3^
*Z* = 8
Mo *K*α radiation
μ = 7.65 mm^−1^
*T* = 296 K
0.22 × 0.12 × 0.10 mm

#### Data collection

Bruker SMART APEXII CCD diffractometer
Absorption correction: multi-scan (*TWINABS*; Bruker, 2005 ▶) *T*_min_ = 0.284, *T*_max_ = 0.515
11292 measured reflections
11292 independent reflections
6212 reflections with *I* > 2σ(*I*)
*R*_int_ = 0.061

#### Refinement

*R*[*F* ^2^ > 2σ(*F* ^2^)] = 0.048
*wR*(*F* ^2^) = 0.101
*S* = 1.01
11292 reflections
546 parameters
H-atom parameters constrained
Δρ_max_ = 1.28 e Å^−3^
Δρ_min_ = −1.11 e Å^−3^

### 

Data collection: *APEX2* (Bruker, 2005 ▶); cell refinement: *SAINT* (Bruker, 2005 ▶); data reduction: *SAINT*; program(s) used to solve structure: *SHELXTL* (Sheldrick, 2008 ▶); program(s) used to refine structure: *SHELXTL*; molecular graphics: *SHELXTL*; software used to prepare material for publication: *publCIF* (Westrip, 2010 ▶) and *PLATON* (Spek, 2009 ▶).

## Supplementary Material

Crystal structure: contains datablock(s) global, I. DOI: 10.1107/S1600536812039785/wm2668sup1.cif

Structure factors: contains datablock(s) I. DOI: 10.1107/S1600536812039785/wm2668Isup2.hkl

Additional supplementary materials:  crystallographic information; 3D view; checkCIF report

## Figures and Tables

**Table 1:** Selected bond lengths (Å)

Mo1—O4	1.697 (4)
Mo1—O3	1.699 (4)
Mo1—O2	1.941 (3)
Mo1—O1	2.080 (3)
Mo1—N1	2.149 (4)
Mo1—N2	2.338 (4)
Mo2—O8	1.692 (4)
Mo2—O7	1.697 (4)
Mo2—O5	1.936 (3)
Mo2—O6	2.081 (3)
Mo2—N4	2.157 (4)
Mo2—N3	2.329 (4)

**Table 2:** Hydrogen-bond geometry (Å, °)

*D*—H⋯*A*	*D*—H	H⋯*A*	*D*⋯*A*	*D*—H⋯*A*
C29—H29*C*⋯Br7^i^	0.96	2.88	3.792 (6)	160

Symmetry code: (i) 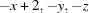.

**Table 3:** C—H⋯π inter­actions (Å,°) *Cg*1 is the centroid of the C24–C29 ring and *Cg*2 is the centroid of the C14–C19 ring.

C—H⋯*Cg*	C—H	H⋯*Cg*	C⋯*Cg*	C—H⋯*Cg*
C12—H12*A*⋯*Cg*1^ii^	0.97	2.73	3.481 (6)	135
C27—H27*A*⋯*Cg*2^iii^	0.97	2.58	3.375 (6)	140

Symmetry codes: (ii) *x*, 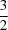 − *y*, −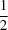 + *z*; (iii) *x*, 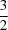 − *y*, 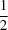 + *z*.

**Fig. 1.**
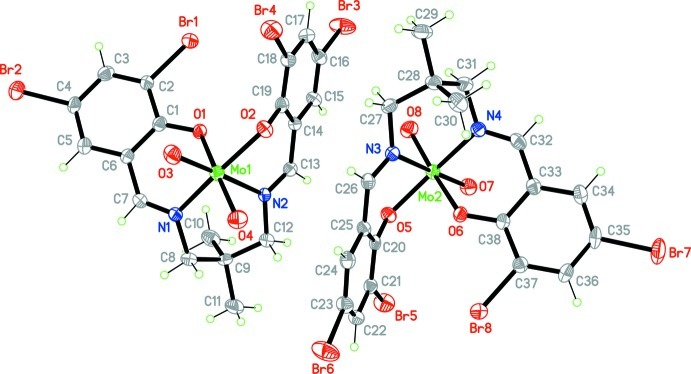
The molecular structure of the title compound, showing 40% probability displacement ellipsoids and the atomic numbering.

**Fig. 2.**
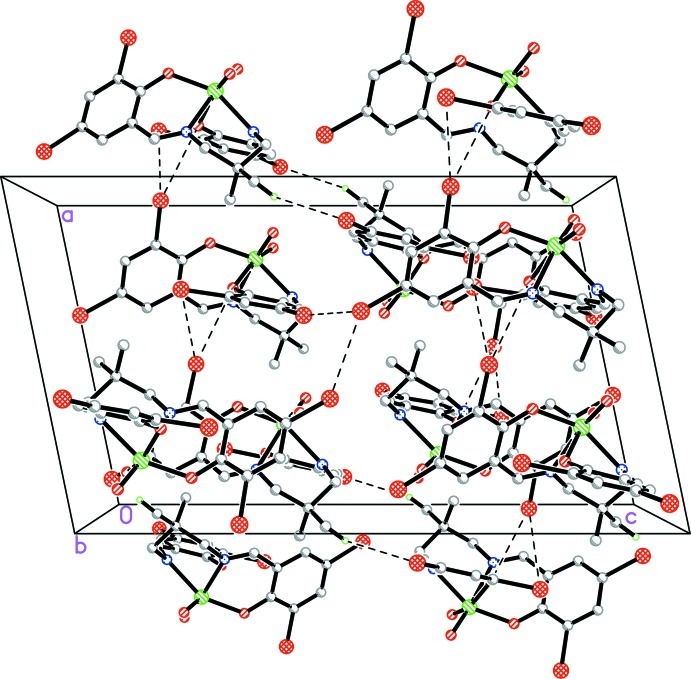
The packing of the complex showing linking of molecules through intermolecular C—H···Br and Br···Br interactions (dashed lines).

## supplementary crystallographic information

### Comment

Molybdenum is unique among the heavier transition metals due to its role as a bio-catalysts in enzymatic reactions in several molybdoproteins (Majumdar & Sarkar, 2011). Therefore the coordination chemistry of molybdenum(VI) has attracted considerable attention due to its biological importance (Enemark *et al.*, 2004; Holm *et al.*, 1996). This element is also applied in various catalytic oxidation reactions (Mancka & Plass, 2007). In continuation of our work on the crystal structure of Schiff base ligands derived from different substituted salicylaldehyde and amine precursors and their complexes (Kargar & Kia, 2011; Kia & Fun, 2009) we determined the crystal structure of the title compound.

The asymmetric unit of the title compound, Fig. 1, comprises two crystallographically independent molcules. For each molecule, the Mo^VI^ atom is coordinated by two oxide O atoms and by two O and two N atoms of the tetradentate Schiff base ligand in a distorted octahedral environment. The dihedral angles between the phenyl rings in the molecules are 76.2 (3) and 77.7 (3)°. The bond lengths and angles are within the normal ranges and comparable to previously reported structures (Abbasi *et al.*, 2008; Monadi *et al.*, 2009). The Mo1—N2 and Mo2—N3 bond lengths *trans* to the terminal oxido groups are significantly longer than the Mo1—N1 and Mo2—N4 bonds, a result attributed to the *trans* effect of the oxido group (Table 1). An interesting feature of the crystal structure are Br···Br contacts [Br3···Br3^iv^ = 3.4420 (17) Å, (iv) 1 - *x*, 2 - *y*, 1 - *z*; Br6···Br6^v^ = 3.5421 (17) Å, (v) -*x*, 2 - *y*, 1 - *z*; Br1···Br5^v^ = 3.6492 (10) Å, (vi) 1 - *x*, 1/2 + *y*, 1/2 - *z*], which are shorter than the sum of the van der Waals radius of Br atoms [3.70 Å] (Bondi, 1964). The crystal structure is further stabilized by intermolecular C—H···Br and C—H···π interactions (Table 2, Fig. 2).

### Experimental

The title dioxidomolybdenum(VI) complex was prepared by mixing MoO_2_(acac)_2_ with the ligand, bis(3,5-dibromosalicylidene)-2,2-dimethyl-1,3-propandiamine, in a 1:1 molar ratio using 50 ml of methanol as solvent, followed by refluxing the solution for 2 h. The small dark-yellow crystals that had formed were filtered off and recrystallized from acetonitrile.

### Refinement

The H atoms were included in calculated positions and treated as riding atoms: C—H = 0.93, 0.97 and 0.96 Å for CH, CH_2_ and CH_3_ H atoms, respectively, with U_iso_(H) = kU_eq_(C), k = 1.2 for CH, CH_2_ and 1.5 for CH_3_. The crystal is a non-merohdral twin with a refined BASF ratio of 0.053 (1)/0.947 (1). The twin matrix, [1.002, 0.00, 0.006; 0.000, -1, 0.000; -0.667, 0.000, -1.002], was obtained by *TWINROTMAT* routine in *PLATON* (Spek, 2009). The highest peak (1.28 e Å^-3^), and deepest hole (-1.11 e Å^-3^), are located 1.00 Å and 0.89 Å from Br6, respectively.

### Crystal data

**Table:**

[Mo(C_19_H_16_Br_4_N_2_O_2_)O_2_]	*F*(000) = 2864
*M**_r_* = 751.92	*D*_x_ = 2.201 Mg m^−^^3^
Monoclinic, *P*2_1_/*c*	Mo *K*α radiation, λ = 0.71073 Å
Hall symbol: -P 2ybc	Cell parameters from 3245 reflections
*a* = 13.1915 (6) Å	θ = 2.6–28.4°
*b* = 15.7890 (8) Å	µ = 7.65 mm^−^^1^
*c* = 22.2514 (13) Å	*T* = 296 K
β = 101.702 (3)°	Block, dark-yellow
*V* = 4538.2 (4) Å^3^	0.22 × 0.12 × 0.10 mm
*Z* = 8	

### Data collection

**Table:**

Bruker SMART APEXII CCD diffractometer	11292 independent reflections
Radiation source: fine-focus sealed tube	6212 reflections with *I* > 2σ(*I*)
Graphite monochromator	*R*_int_ = 0.061
φ and ω scans	θ_max_ = 28.4°, θ_min_ = 1.6°
Absorption correction: multi-scan (TWINABS; Bruker, 2005)	*h* = −17→16
*T*_min_ = 0.284, *T*_max_ = 0.515	*k* = −21→21
11292 measured reflections	*l* = −26→29

### Refinement

**Table:**

Refinement on *F*^2^	Primary atom site location: structure-invariant direct methods
Least-squares matrix: full	Secondary atom site location: difference Fourier map
*R*[*F*^2^ > 2σ(*F*^2^)] = 0.048	Hydrogen site location: inferred from neighbouring sites
*wR*(*F*^2^) = 0.101	H-atom parameters constrained
*S* = 1.01	*w* = 1/[σ^2^(*F*_o_^2^) + (0.0281*P*)^2^ + 6.3956*P*] where *P* = (*F*_o_^2^ + 2*F*_c_^2^)/3
11292 reflections	(Δ/σ)_max_ = 0.001
546 parameters	Δρ_max_ = 1.28 e Å^−^^3^
0 restraints	Δρ_min_ = −1.11 e Å^−^^3^

### Special details

**Table:**

Geometry. All esds (except the esd in the dihedral angle between two l.s. planes) are estimated using the full covariance matrix. The cell esds are taken into account individually in the estimation of esds in distances, angles and torsion angles; correlations between esds in cell parameters are only used when they are defined by crystal symmetry. An approximate (isotropic) treatment of cell esds is used for estimating esds involving l.s. planes.
Refinement. Refinement of F^2^ against ALL reflections. The weighted R-factor wR and goodness of fit S are based on F^2^, conventional R-factors R are based on F, with F set to zero for negative F^2^. The threshold expression of F^2^ > 2sigma(F^2^) is used only for calculating R-factors(gt) etc. and is not relevant to the choice of reflections for refinement. R-factors based on F^2^ are statistically about twice as large as those based on F, and R-factors based on ALL data will be even larger.

### Fractional atomic coordinates and isotropic or equivalent isotropic displacement parameters (Å^2^)

**Table:**

	*x*	*y*	*z*	*U*_iso_*/*U*_eq_	
Mo1	0.80014 (4)	0.41601 (3)	0.37294 (2)	0.03426 (13)	
Br1	0.69649 (5)	0.65616 (4)	0.23848 (3)	0.05079 (18)	
Br2	0.61510 (6)	0.83218 (4)	0.44390 (3)	0.0634 (2)	
Br3	0.62244 (6)	0.47603 (6)	0.03994 (3)	0.0773 (3)	
Br4	0.97307 (5)	0.51887 (5)	0.22847 (3)	0.05783 (19)	
O1	0.7060 (3)	0.5093 (2)	0.32457 (16)	0.0344 (9)	
O2	0.8499 (3)	0.4083 (2)	0.29679 (16)	0.0362 (9)	
O3	0.8767 (3)	0.4894 (3)	0.41598 (17)	0.0484 (11)	
O4	0.8398 (3)	0.3227 (3)	0.40775 (18)	0.0506 (11)	
N1	0.6810 (3)	0.4269 (3)	0.42559 (19)	0.0358 (11)	
N2	0.6677 (3)	0.3345 (3)	0.3147 (2)	0.0318 (10)	
C1	0.6815 (4)	0.5780 (4)	0.3506 (2)	0.0349 (13)	
C2	0.6747 (4)	0.6565 (4)	0.3198 (2)	0.0365 (14)	
C3	0.6547 (4)	0.7295 (4)	0.3464 (3)	0.0427 (15)	
H3	0.6526	0.7803	0.3250	0.051*	
C4	0.6370 (5)	0.7287 (4)	0.4061 (3)	0.0432 (15)	
C5	0.6359 (5)	0.6547 (4)	0.4367 (3)	0.0456 (15)	
H5	0.6199	0.6544	0.4756	0.055*	
C6	0.6587 (4)	0.5783 (3)	0.4099 (3)	0.0363 (13)	
C7	0.6439 (4)	0.4990 (4)	0.4383 (3)	0.0411 (14)	
H7	0.6043	0.4994	0.4684	0.049*	
C8	0.6367 (5)	0.3499 (4)	0.4466 (3)	0.0432 (15)	
H8A	0.6004	0.3646	0.4790	0.052*	
H8B	0.6922	0.3113	0.4637	0.052*	
C9	0.5615 (4)	0.3055 (3)	0.3947 (3)	0.0379 (14)	
C10	0.4717 (4)	0.3638 (4)	0.3669 (3)	0.0535 (17)	
H10A	0.4978	0.4116	0.3481	0.080*	
H10B	0.4378	0.3831	0.3986	0.080*	
H10C	0.4233	0.3333	0.3365	0.080*	
C11	0.5204 (5)	0.2280 (4)	0.4240 (3)	0.062 (2)	
H11A	0.4784	0.1943	0.3926	0.093*	
H11B	0.4795	0.2466	0.4526	0.093*	
H11C	0.5776	0.1948	0.4452	0.093*	
C12	0.6156 (4)	0.2712 (3)	0.3458 (3)	0.0408 (14)	
H12A	0.6664	0.2296	0.3646	0.049*	
H12B	0.5648	0.2420	0.3151	0.049*	
C13	0.6461 (4)	0.3373 (3)	0.2566 (2)	0.0328 (13)	
H13	0.5933	0.3022	0.2365	0.039*	
C14	0.6976 (4)	0.3913 (3)	0.2187 (2)	0.0322 (13)	
C15	0.6479 (4)	0.4054 (4)	0.1582 (2)	0.0383 (14)	
H15	0.5836	0.3810	0.1431	0.046*	
C16	0.6931 (4)	0.4549 (4)	0.1211 (2)	0.0400 (14)	
C17	0.7886 (4)	0.4908 (4)	0.1418 (3)	0.0422 (15)	
H17	0.8183	0.5254	0.1162	0.051*	
C18	0.8396 (4)	0.4747 (4)	0.2012 (3)	0.0356 (13)	
C19	0.7963 (4)	0.4249 (3)	0.2405 (2)	0.0341 (13)	
Mo2	0.70727 (4)	0.13032 (3)	0.13118 (2)	0.03377 (13)	
Br5	0.52741 (5)	0.01796 (5)	0.26881 (3)	0.0595 (2)	
Br6	0.87859 (6)	0.03688 (6)	0.45921 (3)	0.0780 (3)	
Br7	0.89285 (7)	−0.28089 (5)	0.04907 (4)	0.0770 (3)	
Br8	0.79532 (5)	−0.11866 (4)	0.25519 (3)	0.05139 (18)	
O5	0.6550 (3)	0.1341 (2)	0.20641 (16)	0.0375 (9)	
O6	0.7997 (3)	0.0337 (2)	0.17652 (15)	0.0352 (9)	
O7	0.6303 (3)	0.0596 (2)	0.08586 (17)	0.0455 (10)	
O8	0.6704 (3)	0.2251 (2)	0.09839 (18)	0.0486 (11)	
N3	0.8393 (3)	0.2063 (3)	0.1937 (2)	0.0335 (11)	
N4	0.8290 (3)	0.1228 (3)	0.07968 (19)	0.0361 (11)	
C20	0.7057 (4)	0.1136 (3)	0.2630 (2)	0.0311 (13)	
C21	0.6602 (4)	0.0609 (3)	0.2998 (3)	0.0346 (13)	
C22	0.7109 (4)	0.0386 (4)	0.3582 (2)	0.0400 (14)	
H22	0.6800	0.0021	0.3819	0.048*	
C23	0.8070 (4)	0.0707 (4)	0.3806 (3)	0.0424 (15)	
C24	0.8533 (4)	0.1245 (4)	0.3466 (3)	0.0395 (14)	
H24	0.9178	0.1474	0.3634	0.047*	
C25	0.8051 (4)	0.1456 (3)	0.2871 (2)	0.0317 (13)	
C26	0.8593 (4)	0.2006 (3)	0.2517 (2)	0.0338 (13)	
H26	0.9127	0.2339	0.2731	0.041*	
C27	0.8941 (4)	0.2716 (3)	0.1657 (2)	0.0380 (14)	
H27A	0.9438	0.2989	0.1980	0.046*	
H27B	0.8443	0.3143	0.1475	0.046*	
C28	0.9508 (4)	0.2407 (4)	0.1170 (3)	0.0404 (14)	
C29	0.9967 (5)	0.3206 (4)	0.0930 (3)	0.0618 (19)	
H29A	1.0375	0.3509	0.1268	0.093*	
H29B	0.9416	0.3563	0.0723	0.093*	
H29C	1.0396	0.3044	0.0649	0.093*	
C30	1.0372 (4)	0.1792 (4)	0.1436 (3)	0.0540 (17)	
H30A	1.0079	0.1290	0.1575	0.081*	
H30B	1.0832	0.2054	0.1774	0.081*	
H30C	1.0750	0.1643	0.1125	0.081*	
C31	0.8756 (5)	0.2013 (4)	0.0623 (3)	0.0449 (15)	
H31A	0.8212	0.2417	0.0466	0.054*	
H31B	0.9126	0.1892	0.0297	0.054*	
C32	0.8654 (4)	0.0517 (4)	0.0650 (2)	0.0423 (15)	
H32	0.9064	0.0530	0.0356	0.051*	
C33	0.8481 (4)	−0.0292 (4)	0.0903 (3)	0.0391 (14)	
C34	0.8719 (5)	−0.1031 (4)	0.0602 (3)	0.0480 (16)	
H34	0.8906	−0.0999	0.0221	0.058*	
C35	0.8670 (5)	−0.1794 (4)	0.0884 (3)	0.0478 (16)	
C36	0.8449 (4)	−0.1855 (4)	0.1463 (3)	0.0442 (15)	
H36	0.8439	−0.2382	0.1649	0.053*	
C37	0.8244 (4)	−0.1138 (4)	0.1763 (3)	0.0376 (14)	
C38	0.8211 (4)	−0.0338 (3)	0.1483 (2)	0.0328 (13)	

### Atomic displacement parameters (Å^2^)

**Table:**

	*U*^11^	*U*^22^	*U*^33^	*U*^12^	*U*^13^	*U*^23^
Mo1	0.0350 (3)	0.0377 (3)	0.0285 (3)	0.0038 (2)	0.0026 (2)	0.0013 (2)
Br1	0.0725 (5)	0.0421 (4)	0.0404 (4)	−0.0014 (3)	0.0177 (3)	0.0051 (3)
Br2	0.0913 (6)	0.0364 (4)	0.0697 (5)	0.0113 (4)	0.0332 (4)	−0.0066 (3)
Br3	0.0724 (5)	0.1053 (7)	0.0456 (4)	−0.0212 (5)	−0.0082 (4)	0.0336 (4)
Br4	0.0410 (4)	0.0710 (5)	0.0613 (5)	−0.0173 (3)	0.0100 (3)	−0.0028 (4)
O1	0.042 (2)	0.029 (2)	0.031 (2)	0.0061 (18)	0.0062 (17)	0.0011 (17)
O2	0.028 (2)	0.046 (3)	0.034 (2)	0.0075 (17)	0.0056 (17)	0.0007 (18)
O3	0.044 (2)	0.062 (3)	0.037 (2)	−0.011 (2)	0.0017 (18)	−0.008 (2)
O4	0.052 (3)	0.049 (3)	0.046 (3)	0.014 (2)	−0.001 (2)	0.011 (2)
N1	0.048 (3)	0.032 (3)	0.026 (2)	0.001 (2)	0.006 (2)	0.001 (2)
N2	0.038 (3)	0.028 (3)	0.032 (3)	0.002 (2)	0.013 (2)	−0.002 (2)
C1	0.036 (3)	0.031 (3)	0.035 (3)	0.002 (3)	0.003 (3)	−0.001 (3)
C2	0.045 (3)	0.031 (3)	0.034 (3)	0.002 (3)	0.010 (3)	0.000 (3)
C3	0.044 (4)	0.033 (4)	0.052 (4)	0.003 (3)	0.014 (3)	0.005 (3)
C4	0.053 (4)	0.030 (4)	0.050 (4)	0.004 (3)	0.018 (3)	−0.009 (3)
C5	0.057 (4)	0.038 (4)	0.045 (4)	0.001 (3)	0.017 (3)	−0.008 (3)
C6	0.043 (3)	0.027 (3)	0.039 (3)	0.002 (3)	0.012 (3)	−0.001 (3)
C7	0.054 (4)	0.040 (4)	0.032 (3)	−0.002 (3)	0.013 (3)	0.000 (3)
C8	0.057 (4)	0.039 (4)	0.035 (3)	−0.002 (3)	0.014 (3)	0.009 (3)
C9	0.048 (4)	0.028 (3)	0.040 (3)	−0.004 (3)	0.016 (3)	0.006 (3)
C10	0.045 (4)	0.069 (5)	0.048 (4)	0.008 (3)	0.014 (3)	0.010 (3)
C11	0.080 (5)	0.061 (5)	0.053 (4)	−0.021 (4)	0.030 (4)	0.003 (3)
C12	0.050 (4)	0.037 (4)	0.038 (3)	−0.003 (3)	0.015 (3)	0.002 (3)
C13	0.029 (3)	0.033 (3)	0.038 (3)	0.001 (2)	0.008 (2)	−0.001 (2)
C14	0.036 (3)	0.030 (3)	0.033 (3)	0.000 (2)	0.011 (3)	−0.001 (2)
C15	0.039 (3)	0.045 (4)	0.032 (3)	0.001 (3)	0.008 (3)	0.003 (3)
C16	0.040 (3)	0.048 (4)	0.032 (3)	0.005 (3)	0.006 (3)	0.001 (3)
C17	0.050 (4)	0.037 (4)	0.042 (4)	−0.003 (3)	0.014 (3)	0.004 (3)
C18	0.030 (3)	0.038 (4)	0.040 (3)	−0.005 (3)	0.009 (3)	−0.003 (3)
C19	0.034 (3)	0.031 (3)	0.037 (3)	0.009 (3)	0.006 (3)	−0.001 (3)
Mo2	0.0349 (3)	0.0358 (3)	0.0283 (3)	0.0035 (2)	0.0009 (2)	0.0005 (2)
Br5	0.0416 (4)	0.0721 (5)	0.0635 (5)	−0.0158 (3)	0.0073 (3)	0.0017 (4)
Br6	0.0805 (5)	0.1085 (7)	0.0381 (4)	−0.0122 (5)	−0.0043 (4)	0.0208 (4)
Br7	0.0985 (6)	0.0559 (5)	0.0780 (6)	0.0189 (4)	0.0208 (5)	−0.0274 (4)
Br8	0.0708 (5)	0.0417 (4)	0.0442 (4)	0.0001 (3)	0.0176 (3)	0.0039 (3)
O5	0.035 (2)	0.046 (2)	0.031 (2)	0.0027 (18)	0.0050 (17)	0.0014 (18)
O6	0.045 (2)	0.032 (2)	0.027 (2)	0.0060 (18)	0.0024 (17)	−0.0010 (16)
O7	0.047 (2)	0.051 (3)	0.034 (2)	−0.006 (2)	−0.0010 (18)	−0.0021 (19)
O8	0.051 (3)	0.043 (3)	0.048 (3)	0.011 (2)	0.001 (2)	0.0092 (19)
N3	0.036 (3)	0.031 (3)	0.034 (3)	0.002 (2)	0.008 (2)	0.001 (2)
N4	0.043 (3)	0.038 (3)	0.026 (2)	−0.002 (2)	0.002 (2)	0.000 (2)
C20	0.030 (3)	0.029 (3)	0.036 (3)	0.008 (2)	0.012 (3)	−0.006 (2)
C21	0.033 (3)	0.034 (3)	0.039 (3)	0.000 (3)	0.011 (3)	−0.003 (3)
C22	0.045 (4)	0.041 (4)	0.035 (3)	0.001 (3)	0.011 (3)	0.004 (3)
C23	0.045 (4)	0.049 (4)	0.030 (3)	0.005 (3)	0.000 (3)	0.003 (3)
C24	0.036 (3)	0.042 (4)	0.040 (3)	0.001 (3)	0.005 (3)	−0.006 (3)
C25	0.033 (3)	0.035 (3)	0.028 (3)	0.003 (3)	0.006 (2)	−0.001 (2)
C26	0.029 (3)	0.036 (3)	0.036 (3)	0.004 (3)	0.007 (2)	−0.005 (2)
C27	0.045 (3)	0.029 (3)	0.039 (3)	−0.002 (3)	0.006 (3)	0.003 (2)
C28	0.041 (3)	0.043 (4)	0.038 (3)	−0.010 (3)	0.011 (3)	0.002 (3)
C29	0.083 (5)	0.057 (5)	0.050 (4)	−0.024 (4)	0.024 (4)	0.005 (3)
C30	0.047 (4)	0.067 (5)	0.050 (4)	0.002 (4)	0.015 (3)	0.001 (3)
C31	0.059 (4)	0.042 (4)	0.034 (3)	−0.003 (3)	0.012 (3)	0.005 (3)
C32	0.046 (4)	0.055 (4)	0.026 (3)	−0.003 (3)	0.007 (3)	−0.003 (3)
C33	0.041 (3)	0.042 (4)	0.035 (3)	−0.001 (3)	0.008 (3)	−0.005 (3)
C34	0.052 (4)	0.051 (4)	0.043 (4)	0.007 (3)	0.013 (3)	−0.010 (3)
C35	0.047 (4)	0.042 (4)	0.052 (4)	0.009 (3)	0.005 (3)	−0.023 (3)
C36	0.047 (4)	0.032 (4)	0.051 (4)	0.007 (3)	0.003 (3)	−0.006 (3)
C37	0.036 (3)	0.038 (4)	0.037 (3)	0.004 (3)	0.006 (3)	0.000 (3)
C38	0.029 (3)	0.033 (3)	0.036 (3)	0.002 (3)	0.003 (2)	−0.003 (3)

### Geometric parameters (Å, º)

**Table:**

Mo1—O4	1.697 (4)	Mo2—O8	1.692 (4)
Mo1—O3	1.699 (4)	Mo2—O7	1.697 (4)
Mo1—O2	1.941 (3)	Mo2—O5	1.936 (3)
Mo1—O1	2.080 (3)	Mo2—O6	2.081 (3)
Mo1—N1	2.149 (4)	Mo2—N4	2.157 (4)
Mo1—N2	2.338 (4)	Mo2—N3	2.329 (4)
Br1—C2	1.889 (5)	Br5—C21	1.874 (5)
Br2—C4	1.887 (5)	Br6—C23	1.889 (5)
Br3—C16	1.887 (5)	Br7—C35	1.890 (6)
Br4—C18	1.877 (5)	Br8—C37	1.874 (6)
O1—C1	1.301 (6)	O5—C20	1.341 (6)
O2—C19	1.335 (6)	O6—C38	1.297 (6)
N1—C7	1.292 (7)	N3—C26	1.266 (6)
N1—C8	1.466 (7)	N3—C27	1.469 (6)
N2—C13	1.268 (6)	N4—C32	1.290 (7)
N2—C12	1.464 (6)	N4—C31	1.471 (7)
C1—C2	1.409 (7)	C20—C21	1.386 (7)
C1—C6	1.413 (7)	C20—C25	1.405 (7)
C2—C3	1.345 (7)	C21—C22	1.381 (7)
C3—C4	1.395 (8)	C22—C23	1.363 (7)
C3—H3	0.9300	C22—H22	0.9300
C4—C5	1.354 (8)	C23—C24	1.361 (8)
C5—C6	1.405 (7)	C24—C25	1.387 (7)
C5—H5	0.9300	C24—H24	0.9300
C6—C7	1.434 (8)	C25—C26	1.455 (7)
C7—H7	0.9300	C26—H26	0.9300
C8—C9	1.533 (8)	C27—C28	1.516 (7)
C8—H8A	0.9700	C27—H27A	0.9700
C8—H8B	0.9700	C27—H27B	0.9700
C9—C12	1.517 (7)	C28—C30	1.523 (8)
C9—C10	1.528 (8)	C28—C31	1.537 (7)
C9—C11	1.536 (7)	C28—C29	1.541 (8)
C10—H10A	0.9600	C29—H29A	0.9600
C10—H10B	0.9600	C29—H29B	0.9600
C10—H10C	0.9600	C29—H29C	0.9600
C11—H11A	0.9600	C30—H30A	0.9600
C11—H11B	0.9600	C30—H30B	0.9600
C11—H11C	0.9600	C30—H30C	0.9600
C12—H12A	0.9700	C31—H31A	0.9700
C12—H12B	0.9700	C31—H31B	0.9700
C13—C14	1.460 (7)	C32—C33	1.432 (8)
C13—H13	0.9300	C32—H32	0.9300
C14—C15	1.390 (7)	C33—C38	1.410 (7)
C14—C19	1.398 (7)	C33—C34	1.411 (8)
C15—C16	1.360 (8)	C34—C35	1.366 (8)
C15—H15	0.9300	C34—H34	0.9300
C16—C17	1.372 (7)	C35—C36	1.382 (8)
C17—C18	1.380 (7)	C36—C37	1.367 (7)
C17—H17	0.9300	C36—H36	0.9300
C18—C19	1.382 (7)	C37—C38	1.404 (7)
			
O4—Mo1—O3	103.99 (19)	O8—Mo2—O7	103.94 (19)
O4—Mo1—O2	102.49 (17)	O8—Mo2—O5	102.94 (17)
O3—Mo1—O2	105.50 (17)	O7—Mo2—O5	105.01 (17)
O4—Mo1—O1	161.28 (17)	O8—Mo2—O6	161.06 (17)
O3—Mo1—O1	91.90 (17)	O7—Mo2—O6	91.52 (16)
O2—Mo1—O1	82.25 (14)	O5—Mo2—O6	83.09 (14)
O4—Mo1—N1	90.71 (18)	O8—Mo2—N4	89.96 (18)
O3—Mo1—N1	93.14 (18)	O7—Mo2—N4	93.90 (18)
O2—Mo1—N1	153.52 (15)	O5—Mo2—N4	153.52 (15)
O1—Mo1—N1	78.43 (15)	O6—Mo2—N4	77.96 (15)
O4—Mo1—N2	84.34 (17)	O8—Mo2—N3	85.22 (17)
O3—Mo1—N2	168.34 (17)	O7—Mo2—N3	168.14 (17)
O2—Mo1—N2	80.14 (15)	O5—Mo2—N3	79.74 (15)
O1—Mo1—N2	78.61 (14)	O6—Mo2—N3	78.15 (14)
N1—Mo1—N2	78.44 (15)	N4—Mo2—N3	78.40 (16)
C1—O1—Mo1	122.4 (3)	C20—O5—Mo2	127.4 (3)
C19—O2—Mo1	126.8 (3)	C38—O6—Mo2	122.1 (3)
C7—N1—C8	117.8 (5)	C26—N3—C27	117.6 (5)
C7—N1—Mo1	122.8 (4)	C26—N3—Mo2	123.2 (4)
C8—N1—Mo1	119.4 (4)	C27—N3—Mo2	118.8 (3)
C13—N2—C12	118.3 (5)	C32—N4—C31	118.1 (5)
C13—N2—Mo1	122.4 (4)	C32—N4—Mo2	122.6 (4)
C12—N2—Mo1	119.0 (3)	C31—N4—Mo2	119.3 (4)
O1—C1—C2	121.0 (5)	O5—C20—C21	120.4 (5)
O1—C1—C6	122.5 (5)	O5—C20—C25	121.4 (5)
C2—C1—C6	116.5 (5)	C21—C20—C25	118.2 (5)
C3—C2—C1	122.6 (5)	C22—C21—C20	121.5 (5)
C3—C2—Br1	120.3 (4)	C22—C21—Br5	119.5 (4)
C1—C2—Br1	117.1 (4)	C20—C21—Br5	119.0 (4)
C2—C3—C4	119.8 (6)	C23—C22—C21	119.1 (5)
C2—C3—H3	120.1	C23—C22—H22	120.5
C4—C3—H3	120.1	C21—C22—H22	120.5
C5—C4—C3	120.6 (5)	C24—C23—C22	121.2 (5)
C5—C4—Br2	120.3 (5)	C24—C23—Br6	119.7 (4)
C3—C4—Br2	119.1 (5)	C22—C23—Br6	119.1 (5)
C4—C5—C6	120.1 (6)	C23—C24—C25	120.6 (5)
C4—C5—H5	120.0	C23—C24—H24	119.7
C6—C5—H5	120.0	C25—C24—H24	119.7
C5—C6—C1	120.3 (5)	C24—C25—C20	119.3 (5)
C5—C6—C7	120.2 (5)	C24—C25—C26	118.6 (5)
C1—C6—C7	118.9 (5)	C20—C25—C26	122.1 (5)
N1—C7—C6	125.5 (5)	N3—C26—C25	124.8 (5)
N1—C7—H7	117.2	N3—C26—H26	117.6
C6—C7—H7	117.2	C25—C26—H26	117.6
N1—C8—C9	112.2 (4)	N3—C27—C28	115.7 (4)
N1—C8—H8A	109.2	N3—C27—H27A	108.4
C9—C8—H8A	109.2	C28—C27—H27A	108.4
N1—C8—H8B	109.2	N3—C27—H27B	108.4
C9—C8—H8B	109.2	C28—C27—H27B	108.4
H8A—C8—H8B	107.9	H27A—C27—H27B	107.4
C12—C9—C10	111.3 (5)	C27—C28—C30	111.5 (5)
C12—C9—C8	112.2 (5)	C27—C28—C31	111.4 (5)
C10—C9—C8	111.0 (5)	C30—C28—C31	111.2 (5)
C12—C9—C11	106.2 (5)	C27—C28—C29	105.7 (5)
C10—C9—C11	109.9 (5)	C30—C28—C29	109.9 (5)
C8—C9—C11	105.9 (5)	C31—C28—C29	107.0 (5)
C9—C10—H10A	109.5	C28—C29—H29A	109.5
C9—C10—H10B	109.5	C28—C29—H29B	109.5
H10A—C10—H10B	109.5	H29A—C29—H29B	109.5
C9—C10—H10C	109.5	C28—C29—H29C	109.5
H10A—C10—H10C	109.5	H29A—C29—H29C	109.5
H10B—C10—H10C	109.5	H29B—C29—H29C	109.5
C9—C11—H11A	109.5	C28—C30—H30A	109.5
C9—C11—H11B	109.5	C28—C30—H30B	109.5
H11A—C11—H11B	109.5	H30A—C30—H30B	109.5
C9—C11—H11C	109.5	C28—C30—H30C	109.5
H11A—C11—H11C	109.5	H30A—C30—H30C	109.5
H11B—C11—H11C	109.5	H30B—C30—H30C	109.5
N2—C12—C9	115.4 (4)	N4—C31—C28	111.7 (4)
N2—C12—H12A	108.4	N4—C31—H31A	109.3
C9—C12—H12A	108.4	C28—C31—H31A	109.3
N2—C12—H12B	108.4	N4—C31—H31B	109.3
C9—C12—H12B	108.4	C28—C31—H31B	109.3
H12A—C12—H12B	107.5	H31A—C31—H31B	108.0
N2—C13—C14	125.0 (5)	N4—C32—C33	125.5 (6)
N2—C13—H13	117.5	N4—C32—H32	117.3
C14—C13—H13	117.5	C33—C32—H32	117.3
C15—C14—C19	119.8 (5)	C38—C33—C34	120.9 (6)
C15—C14—C13	117.9 (5)	C38—C33—C32	119.7 (5)
C19—C14—C13	122.1 (5)	C34—C33—C32	118.8 (6)
C16—C15—C14	120.1 (5)	C35—C34—C33	118.4 (6)
C16—C15—H15	120.0	C35—C34—H34	120.8
C14—C15—H15	120.0	C33—C34—H34	120.8
C15—C16—C17	121.4 (5)	C34—C35—C36	121.9 (6)
C15—C16—Br3	119.4 (4)	C34—C35—Br7	120.4 (5)
C17—C16—Br3	119.3 (4)	C36—C35—Br7	117.7 (5)
C16—C17—C18	118.7 (5)	C37—C36—C35	119.8 (6)
C16—C17—H17	120.7	C37—C36—H36	120.1
C18—C17—H17	120.7	C35—C36—H36	120.1
C17—C18—C19	121.8 (5)	C36—C37—C38	121.4 (6)
C17—C18—Br4	119.0 (4)	C36—C37—Br8	121.4 (5)
C19—C18—Br4	119.2 (4)	C38—C37—Br8	117.2 (4)
O2—C19—C18	119.9 (5)	O6—C38—C37	121.1 (5)
O2—C19—C14	122.0 (5)	O6—C38—C33	121.4 (5)
C18—C19—C14	118.2 (5)	C37—C38—C33	117.4 (5)
			
O4—Mo1—O1—C1	110.4 (6)	O8—Mo2—O5—C20	−132.9 (4)
O3—Mo1—O1—C1	−38.0 (4)	O7—Mo2—O5—C20	118.5 (4)
O2—Mo1—O1—C1	−143.4 (4)	O6—Mo2—O5—C20	28.8 (4)
N1—Mo1—O1—C1	54.8 (4)	N4—Mo2—O5—C20	−15.6 (7)
N2—Mo1—O1—C1	135.2 (4)	N3—Mo2—O5—C20	−50.3 (4)
O4—Mo1—O2—C19	132.3 (4)	O8—Mo2—O6—C38	−108.5 (6)
O3—Mo1—O2—C19	−119.2 (4)	O7—Mo2—O6—C38	36.6 (4)
O1—Mo1—O2—C19	−29.3 (4)	O5—Mo2—O6—C38	141.5 (4)
N1—Mo1—O2—C19	14.0 (7)	N4—Mo2—O6—C38	−57.1 (4)
N2—Mo1—O2—C19	50.4 (4)	N3—Mo2—O6—C38	−137.6 (4)
O4—Mo1—N1—C7	153.6 (4)	O8—Mo2—N3—C26	129.6 (4)
O3—Mo1—N1—C7	49.5 (5)	O7—Mo2—N3—C26	−89.3 (9)
O2—Mo1—N1—C7	−85.8 (6)	O5—Mo2—N3—C26	25.5 (4)
O1—Mo1—N1—C7	−41.8 (4)	O6—Mo2—N3—C26	−59.6 (4)
N2—Mo1—N1—C7	−122.3 (4)	N4—Mo2—N3—C26	−139.5 (4)
O4—Mo1—N1—C8	−28.5 (4)	O8—Mo2—N3—C27	−42.9 (4)
O3—Mo1—N1—C8	−132.5 (4)	O7—Mo2—N3—C27	98.3 (9)
O2—Mo1—N1—C8	92.2 (5)	O5—Mo2—N3—C27	−147.0 (4)
O1—Mo1—N1—C8	136.2 (4)	O6—Mo2—N3—C27	128.0 (4)
N2—Mo1—N1—C8	55.6 (4)	N4—Mo2—N3—C27	48.1 (4)
O4—Mo1—N2—C13	−129.3 (4)	O8—Mo2—N4—C32	−153.0 (4)
O3—Mo1—N2—C13	94.4 (9)	O7—Mo2—N4—C32	−49.0 (4)
O2—Mo1—N2—C13	−25.6 (4)	O5—Mo2—N4—C32	87.0 (6)
O1—Mo1—N2—C13	58.4 (4)	O6—Mo2—N4—C32	41.7 (4)
N1—Mo1—N2—C13	138.8 (4)	N3—Mo2—N4—C32	121.9 (4)
O4—Mo1—N2—C12	43.7 (4)	O8—Mo2—N4—C31	29.4 (4)
O3—Mo1—N2—C12	−92.6 (9)	O7—Mo2—N4—C31	133.3 (4)
O2—Mo1—N2—C12	147.4 (4)	O5—Mo2—N4—C31	−90.7 (5)
O1—Mo1—N2—C12	−128.6 (4)	O6—Mo2—N4—C31	−135.9 (4)
N1—Mo1—N2—C12	−48.2 (4)	N3—Mo2—N4—C31	−55.8 (4)
Mo1—O1—C1—C2	142.0 (4)	Mo2—O5—C20—C21	−131.8 (4)
Mo1—O1—C1—C6	−39.2 (7)	Mo2—O5—C20—C25	49.1 (7)
O1—C1—C2—C3	−176.6 (5)	O5—C20—C21—C22	179.7 (5)
C6—C1—C2—C3	4.5 (8)	C25—C20—C21—C22	−1.2 (8)
O1—C1—C2—Br1	2.7 (7)	O5—C20—C21—Br5	0.6 (7)
C6—C1—C2—Br1	−176.2 (4)	C25—C20—C21—Br5	179.7 (4)
C1—C2—C3—C4	−1.7 (9)	C20—C21—C22—C23	1.5 (8)
Br1—C2—C3—C4	178.9 (4)	Br5—C21—C22—C23	−179.3 (4)
C2—C3—C4—C5	−2.6 (9)	C21—C22—C23—C24	0.3 (9)
C2—C3—C4—Br2	177.2 (4)	C21—C22—C23—Br6	−177.6 (4)
C3—C4—C5—C6	3.9 (9)	C22—C23—C24—C25	−2.4 (9)
Br2—C4—C5—C6	−175.9 (4)	Br6—C23—C24—C25	175.5 (4)
C4—C5—C6—C1	−1.0 (9)	C23—C24—C25—C20	2.7 (8)
C4—C5—C6—C7	−171.5 (6)	C23—C24—C25—C26	−177.5 (5)
O1—C1—C6—C5	178.0 (5)	O5—C20—C25—C24	178.2 (5)
C2—C1—C6—C5	−3.1 (8)	C21—C20—C25—C24	−0.9 (8)
O1—C1—C6—C7	−11.3 (8)	O5—C20—C25—C26	−1.6 (8)
C2—C1—C6—C7	167.6 (5)	C21—C20—C25—C26	179.3 (5)
C8—N1—C7—C6	−164.2 (5)	C27—N3—C26—C25	173.6 (5)
Mo1—N1—C7—C6	13.8 (8)	Mo2—N3—C26—C25	1.1 (7)
C5—C6—C7—N1	−165.1 (6)	C24—C25—C26—N3	159.7 (5)
C1—C6—C7—N1	24.2 (9)	C20—C25—C26—N3	−20.5 (8)
C7—N1—C8—C9	102.4 (6)	C26—N3—C27—C28	126.3 (5)
Mo1—N1—C8—C9	−75.6 (5)	Mo2—N3—C27—C28	−60.9 (6)
N1—C8—C9—C12	65.7 (6)	N3—C27—C28—C30	−63.3 (6)
N1—C8—C9—C10	−59.6 (6)	N3—C27—C28—C31	61.6 (6)
N1—C8—C9—C11	−178.8 (5)	N3—C27—C28—C29	177.4 (5)
C13—N2—C12—C9	−126.7 (5)	C32—N4—C31—C28	−101.6 (6)
Mo1—N2—C12—C9	60.1 (6)	Mo2—N4—C31—C28	76.1 (5)
C10—C9—C12—N2	64.8 (6)	C27—C28—C31—N4	−66.6 (6)
C8—C9—C12—N2	−60.4 (6)	C30—C28—C31—N4	58.5 (6)
C11—C9—C12—N2	−175.6 (5)	C29—C28—C31—N4	178.5 (5)
C12—N2—C13—C14	−174.3 (5)	C31—N4—C32—C33	164.0 (5)
Mo1—N2—C13—C14	−1.2 (7)	Mo2—N4—C32—C33	−13.7 (8)
N2—C13—C14—C15	−162.1 (5)	N4—C32—C33—C38	−23.1 (9)
N2—C13—C14—C19	21.1 (8)	N4—C32—C33—C34	165.3 (5)
C19—C14—C15—C16	−2.8 (8)	C38—C33—C34—C35	0.8 (9)
C13—C14—C15—C16	−179.7 (5)	C32—C33—C34—C35	172.3 (5)
C14—C15—C16—C17	0.9 (9)	C33—C34—C35—C36	−3.5 (9)
C14—C15—C16—Br3	−178.1 (4)	C33—C34—C35—Br7	177.5 (4)
C15—C16—C17—C18	1.2 (9)	C34—C35—C36—C37	1.8 (9)
Br3—C16—C17—C18	−179.8 (4)	Br7—C35—C36—C37	−179.2 (4)
C16—C17—C18—C19	−1.4 (9)	C35—C36—C37—C38	2.6 (9)
C16—C17—C18—Br4	176.8 (4)	C35—C36—C37—Br8	−179.3 (4)
Mo1—O2—C19—C18	133.1 (4)	Mo2—O6—C38—C37	−140.7 (4)
Mo1—O2—C19—C14	−49.0 (7)	Mo2—O6—C38—C33	43.2 (6)
C17—C18—C19—O2	177.4 (5)	C36—C37—C38—O6	178.7 (5)
Br4—C18—C19—O2	−0.7 (7)	Br8—C37—C38—O6	0.6 (7)
C17—C18—C19—C14	−0.5 (8)	C36—C37—C38—C33	−5.1 (8)
Br4—C18—C19—C14	−178.7 (4)	Br8—C37—C38—C33	176.8 (4)
C15—C14—C19—O2	−175.3 (5)	C34—C33—C38—O6	179.6 (5)
C13—C14—C19—O2	1.4 (8)	C32—C33—C38—O6	8.2 (8)
C15—C14—C19—C18	2.6 (8)	C34—C33—C38—C37	3.3 (8)
C13—C14—C19—C18	179.3 (5)	C32—C33—C38—C37	−168.0 (5)

### Hydrogen-bond geometry (Å, º)

**Table:**

*D*—H···*A*	*D*—H	H···*A*	*D*···*A*	*D*—H···*A*
C29—H29*C*···Br7^i^	0.96	2.88	3.792 (6)	160

Symmetry code: (i) −*x*+2, −*y*, −*z*.

### C—H···π interactions (Å,°)

Cg1 is the centroid of the C24–C29 ring and Cg2 is the centroid of the C14–C19 ring.

**Table:**

C—H···Cg	C—H	H···Cg	C···Cg	C—H···Cg
C12—H12A···*Cg*1^ii^	0.97	2.73	3.481 (6)	135
C27—H27A···*Cg*2^iii^	0.97	2.58	3.375(s.u.?)	140

Symmetry codes: (ii) x, 3/2 - y, -1/2 + z; (iii) x, 3/2 - y, 1/2 + z.
